# Impacts of Food Fortification on Micronutrient Intake and Nutritional Status of Women of Reproductive Age in Africa—A Narrative Review

**DOI:** 10.1016/j.advnut.2025.100463

**Published:** 2025-06-11

**Authors:** Justine B Coomson, Nick W Smith, Warren McNabb

**Affiliations:** Sustainable Nutrition Initiative, Riddet Institute, Massey University, Palmerston North, New Zealand

**Keywords:** hidden hunger, sustainable healthy diets, dietary adequacy, nutritional status, food fortification, acceptability

## Abstract

More than two-thirds of women of reproductive age (WRA) in Africa are estimated to be micronutrient deficient. This is largely due to the widespread poor dietary quality and inadequate intakes of nutrient-dense foods to meet the heightened requirements for WRA. Food fortification is a cost-effective and highly recommended food-based approach for addressing these micronutrient deficiencies in low-income settings like Africa. The strategy has been implemented at different scales within the region for over 3 decades. We conducted a review to find evidence of the impact of food fortification implemented at various scales and across different population circumstances in Africa. We also sought to understand what factors may limit the impact of ongoing fortification programs on micronutrient status. We also explored findings regarding the knowledge and acceptability of fortified foods within the African population as a further barrier to the impact of food fortification on nutritional status. We found that fortification with iron and vitamin A was associated with the most variable impact from targeted and large-scale fortification programs. However, significant positive effects on nutritional status and serum biomarkers were found for food fortification with folate, iodine, and zinc among African women. Generally, fortified foods are acceptable to consumers; however, surveys assessing knowledge and preference for fortified foods found that WRA know little about food fortification and its benefits. Poor coverage of fortification, lower levels of fortificants than are recommended, and use of non-World Health Organization recommended fortificants limit the impact of food fortification on micronutrient intakes and status among WRA in Africa.

**Statement of significance**

This review highlights the potential for food fortification to improve the intake of essential and commonly lacking micronutrients among women of reproductive age (WRA) and what factors currently limit this potential in the African region where the highest burden of global micronutrient deficiency among WRA occurs.

## Introduction

An adequate intake of food macro- and micronutrients is essential for maintaining the health, growth, and development of the human body. However, meeting nutrient needs through the daily diet remains a challenge for over 800 million people globally who are chronically hungry and do not have access to enough calories and for >4 billion people who have inadequate micronutrient intakes and are affected by micronutrient deficiencies [[1], [2], [3]]. Micronutrient deficiency (MND) is one form of malnutrition that impacts over half of the global population and is estimated to have increased significantly across countries of all income classifications in the past 3 decades [4,5].

MND causes morbidity and mortality in individuals and affects human potential with severe consequences including increased susceptibility to infections, birth defects, and impaired cognitive abilities [6,7]. Due to their relatively higher micronutrient requirements for performing physiological functions, women of reproductive age (WRA), children under 5 y, adolescent girls and pregnant and lactating women are the most susceptible to MND and bear the greatest burden globally [4].

The rising prevalence of MND has been blamed on the failure of current diets to provide adequate density of essential micronutrients like iron, vitamin A, folate, and zinc [8]. In low- and middle-income countries (LMIC), the monotonous consumption of nutrient-poor staple foods limits adequate micronutrient intakes among the greater proportion of the population, particularly those in Africa and South Asia [9,10]. Sub-Saharan Africa, for example, accounts for more than half of the global cases of MNDs with an estimated 80% of WRA and 62% of children under-5 being deficient in ≥1 micronutrient [4,11]. In addition, the region also bears the highest prevalence of stunting and wasting among children under 5 worldwide, against a backdrop of rapid population growth [12,13].

To address this growing burden of MNDs in Africa, efficient and sustainable approaches are required. Food-based strategies, particularly large-scale food fortification (LSFF), have been highlighted by the World Health Assembly resolution in 2023 as an important and highly recommended intervention for addressing poor micronutrient intakes [14]. Redesigning food fortification efforts in developing countries promises to enhance their impact toward addressing the high prevalence of hidden hunger [13].

Food fortification has been widely implemented in many African countries and the broader developing regions of the world for over 3 decades. However, the trends in MND within these regions have not seen any significant decline [15]. This static trend questions the effectiveness of existing fortification programs and how they impact nutrient intakes and nutritional status of key vulnerable groups. We sought to provide evidence of the impact of food fortification implemented at different scales and across different population circumstances in Africa and to understand what factors may be limiting the impact of programs on micronutrient status. To do this, we reviewed available research on the impact of food fortification in Africa on nutritional status and prevalence of MND among WRA. Second, we explored findings on the knowledge and acceptability of fortified foods within the African population.

## Methods

This review followed a structured approach to evaluate the available literature on the impact of consuming fortified foods and condiments through mandatory, voluntary, or targeted fortification approaches on nutrient intakes and nutritional outcomes of WRA in Africa.

### Literature search

We searched Scopus, Discover, PubMed, and Google Scholar between April and May 2024 to retrieve published literature on primary studies or studies using secondary data to assess the effects of any form of food or condiment fortification on micronutrient intakes, micronutrient status, and health outcomes or disease biomarkers among WRA in Africa. We included studies conducted between 2000 and 2024, a period within which majority of country-level food fortification programs were implemented in Africa [16]. Databases were searched using the following search terms: (“Food fortification” OR “mandatory fortification” OR “fortification program” OR “fortification”) AND (“Micronutrient intake” OR “nutrient intake” OR “Vitamin” OR “mineral” OR “consumption” OR “requirement” OR “micronutrient deficiency” OR “anemia” OR “iron” OR “vitamin A” OR “iodine” OR “folate” OR “folic acid” OR “iodine” OR “zinc”) AND (“woman” OR “women” “female” OR adult∗ OR “women of reproductive age”) AND (Africa OR sub-Saharan Africa). Iron, iodine, zinc, folate, and vitamin A were included in the search because they are common deficient micronutrients and are often included as fortificants to staple foods in Africa.

### Inclusion criteria for search articles

**FIGURE 1:**
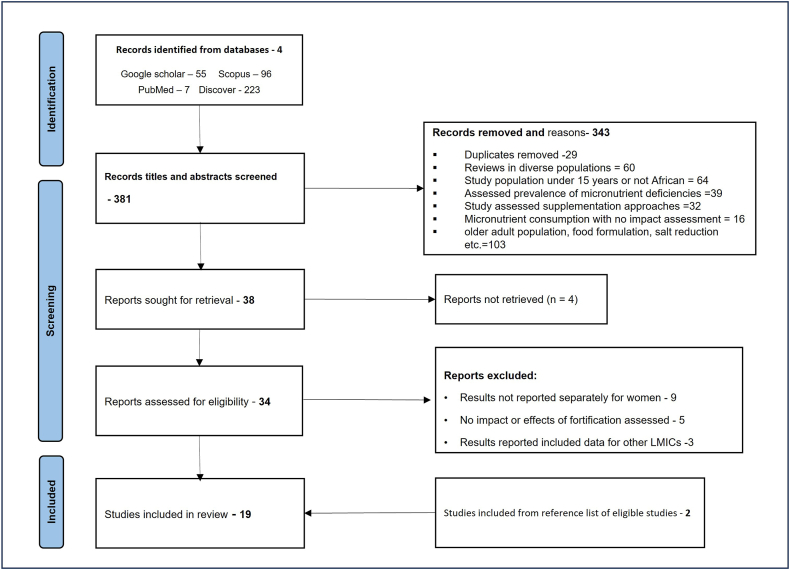
PRISMA flow diagram of the selection of records for review. LMICs, low- and middle-income countries.

Studies were included if they assessed the influence of consumption of fortified foods and condiments (salt, bouillon) on micronutrient status or disease outcome among WRA. Studies were also included if they were based on a population of African women or used data from Africa. If a study included other LMIC but reported the outcome for African women separately, it was considered for inclusion. Studies that estimated the changes in nutritional status and prevalence of MNDs based on estimates of consumption of fortified foods in the population were also included (Figure 1).

Outcomes on nutritional status reported by studies were not restricted to those for the 5 commonly deficient micronutrients. We included studies that examined the effects of fortification on micronutrient intake adequacy and in relation to requirements or addressing deficiency markers. We focused on common MND or disorders such as iron deficiency, anemia, vitamin A deficiency and night blindness, neural tube defects, goiter, and iodine deficiency disorders.

### Screening and inclusion of articles

The literature search from the 4 databases yielded 381 articles. The titles and abstracts of these articles were screened to identify studies that were relevant to food fortification among WRA. The articles excluded at this stage of the screening were 319; they assessed the effect of supplementation and other food- or diet-based approaches on nutrient intakes and studies among other age and population groups as shown in Figure 1. As a secondary interest, studies that assessed women’s knowledge and acceptability of fortification and fortified foods in Africa were also included in the selection. Nineteen articles were included in the final review, comprising primary intervention and observational studies and studies that modeled the changes in health and nutrition outcomes of WRA after fortification based on secondary data.

We examined the full-text records of the included studies, extracting the author names, year of publication, sample size (where applicable), location of study, age group of WRA studied, methods used, indicators of nutrient intakes or impact of fortification assessed, and key findings of the studies and gaps or recommendations. Details of the studies included are reported in TABLE 1, TABLE 2, TABLE 3 [[17], [18], [19], [20], [21], [22], [23], [24], [25], [26], [27], [28], [29], [30], [31], [32], [33], [34], [35], [36]]. Table 1 summarizes the studies that assess the impact of fortification on nutritional status. TABLE 2, TABLE 3 summarize studies that assess the acceptability and knowledge of fortified foods among WRA, respectively.

**TABLE 1:** Studies assessing the impact of fortification on nutrient intake and nutritional status of women of reproductive age in Africa.

Study reference	Population, sample size and location	Study design	Interventions and assessments	Outcome assessment	Findings	Outcome of fortification	Limitations
Iron and vitamin A							
Azupogo et al., 2024 [17]	621 nonpregnant adolescent girls, 10–17 y from a rural district of the northern region of Ghana	A 26-wk double-blind, randomized controlled trial	Provided multiple micronutrient-fortified biscuits (MMB), enriched with 11 vitamins and 7 minerals to an intervention group of adolescents and unfortified biscuits to a control group of girls.	Plasma ferritin (PF), soluble transferrin receptor (sTfR), retinol-binding protein (RBP) (μmol/L), and inflammation biomarkers.	MMB consumption did not improve Hb and iron status but reduced the prevalence of deficient vitamin A status among postmenarche girls. There were no apparent variations in PF, sTfR, or RBP between intervention and control groups.	No effect of MMB was observed on nutritional biomarkers for iron and vitamin A status in the intervention group.	Difference in Hb measurement at baseline and endline might have caused a systematic overestimation of baseline Hb levels
Makola et al., 2003 [18]	259 nonpregnant women; 127 experimental and 132 placebo group from Tanzania with mean age =25.2 y.	Randomized, placebo-controlled double-blind effectiveness trial.	Compared serum biomarkers between an intervention group receiving a micronutrient-fortified beverage fortified with 11 micronutrients and a control group consuming unfortified beverages, respectively, over 8 wk	Hemoglobin concentration, serum ferritin, retinol, and thyroid-stimulating hormone measured at baseline and at the end of 8 wk of intervention	The supplement resulted in a 4.16 g/L increase in Hb and a 3 μg/L increase in ferritin. Risk of anemia and iron deficiency anemia reduced by 51% and 56%, respectively. No differences in retinol concentration for fortified-beverage or placebo groups were observed at the end of the 8 wk.	Iron fortification improved iron status and decreased risk of iron deficiency anemia.	The analysis excluded women with Hb <80 g/L (*n* =36) who would have had a greater potential to benefit from the intervention. The study did not control the effects of inflammation on hemoglobin.
Engle-Stone et al., 2017 [19]	300 nonpregnant women of reproductive age (15–49 y) from Yaoundé and Douala, Cameroon.	Cross-sectional, cluster surveys conducted in 2009 and 2012.	Used pre- and postfortification surveys to compare nutrient intake and status from 2 y before and 1 y after the introduction of fortified wheat flour.	Indicators of inflammation, malaria, anemia, and micronutrient status [plasma ferritin, soluble transferrin receptor (sTfR), zinc, folate, and vitamin B_12_].	Only 34% of wheat flour samples were adequately fortified (Fe ≥60 mg/kg and total Zn ≥95 mg/kg). The prevalence of inadequate iron intake decreased from 85% to 66%, zinc from 40% to 13%, and folate from 79% to 13% after fortification. Maternal anemia prevalence was lower (39.1%) postfortification compared with (46.7%) before fortification. Prevalence of low plasma zinc concentration decreased from 39% before to 21% after fortification	Fortification was associated with higher iron and zinc, status 1-y postfortification	The pre–post survey design limits inferences about causality.
Papathakis and Pearson, 2012 [20]	142 Breast-feeding women (94 HIV-infected, 48 HIV-uninfected), South Africa, Mean age (y) – subsample =24.6, rest of cohort 26.0	A prospective cohort study compared nutrient intakes of women with only prefortification data taken at different times postpartum with a subsample (*n* = 34) of women who had both pre- and postfortification intake data.	Compared nutrient intakes from 4 dietary recalls and serum micronutrient status for pre- and postfortification periods.	Change in mean serum concentrations of retinol, folate, ferritin and zinc between pre- and postfortification periods	Serum folate and zinc increased significantly postfortification for the subsample (*P* <0. 001), no change in ferritin was observed. Postfortification deficient folate status was reduced (73.5 % pre- to 3.0 % -post; *P* <0.001). Zinc deficiency reduced (pre- 26.5% vs. post -5.9%). There was no change in iron deficiency (pre-16.7% vs. post-19.4%)	Fortification is associated with positive effects on folate and zinc status.	The analysis was limited to women with both food records and serum analysis data resulting in a small sample size. The study did not clarify the time points within which fortification began.
Steyn et al., 2008 [21]	1726 nonpregnant women ≥15 y in South Africa	A secondary data analysis of dietary surveys among adults, substituting unfortified foods with fortified options.	Assessed nutrient content of market samples of fortified foods and determined change in nutrient intake with and without fortification	Mean nutrient intakes and nutrient adequacy ratio with/without fortification	Without fortification, calcium and iron intakes were below 50% RNI, and folate and vitamin B_6_ intakes were <75% RNI. Zinc, vitamins A, B_12_, and calcium intakes met the RNI for WRA. With fortification, the MAR increased for women from 59.9% to 72.7% and increased the proportion of requirements met to 100% for all nutrients except iron.	Fortification is associated with a higher mean adequacy ratio for all nutrients fortified except iron.	The secondary data was based on a combined database of dietary surveys done across different time periods and may not be representative of the current nutrient intake status of the population.
Rondini et al., 2022 [22]	90,797 nonpregnant WRA (15–49 y) from 9 African countries—Benin, Cameroon, Ghana, Guinea, Mali, Niger, Senegal, Tanzania, Uganda.	An analytic study of DHS data from countries with mandatory fortification compared with countries with no fortification regulations between 2000 and 2018.	This study used a difference-in-differences approach to compare changes in the prevalence of anemia over time among WRA in countries with mandatory food fortification compared with countries with no mandatory food fortification.	Change in anemia prevalence between 2000 and 2018	Countries with fortification showed 27% decreased odds of anemia (adjusted OR: 0.73; 95% CI: 0.63, 0.85) and a 7.47% decrease in the mean anemia prevalence (average marginal effect: −7.47; 95% CI: −11.03, −3.92) from the pre- to postfortification period	Mandatory fortification of grains was associated with a lower prevalence of anemia.	Potential for residual confounding due to unmeasured factors, such as biomarkers of inflammation which could have biased the findings in either direction.
Rohner et al., 2016 [23]	379 nonpregnant WRA (15–49 y) from 26 clusters each located in central Côte d’Ivoire and Abidjan	A cross-sectional cluster study comparing nutritional status of WRA living in a rural area or urban area based on nutrient contents of fortified food from the 2 settings.	This study compared micronutrient content of fortified foods, consumption levels and prevalence of anemia and Hb concentrations between rural and urban dwelling WRA based on household consumption of fortified oil and wheat flour from the mandatory fortification program.	Assessed hemoglobin (Hb), retinol-binding protein (RBP), ferritin, soluble transferrin receptors (sTfR), subclinical inflammation, and plasmodium spp. infection biomarkers of study sample.	Significant differences in the proportion of adequately fortified wheat flour with iron between the rural (47%) and urban areas (93%). Mean Hb concentrations were higher among urban than rural WRA. More than 3-quarters of rural WRA were anemic compared with less than half of the urban sample.	Low iron fortification of flour was associated with higher anemia prevalence among rural women.	Study did not estimate intakes of fortified flour to compare with status among the women.
Friesen et al., 2020 [24]	Households and nonpregnant WRA (15–49 y) Nigeria, South Africa, Tanzania, and Uganda	Cross-sectional, clustered household surveys of fortified food consumption by WRA.	Compared the estimated nutrient intakes and the proportion of nutrient EAR met using measured and potential fortification levels of mandatory fortified foods for WRA	Estimated daily iron, vitamin A, and iodine intakes from fortified foods, and the proportion of nutrient requirements provided for WRA.	Fortified foods made modest contributions to measured iron intakes (0%–13% RNI); contributed substantially to vitamin A and iodine intakes (20%–125% and 88%–253% EAR, respectively). RNI taken from WHO and FAO. (12% iron bioavailability was assumed in all countries); EAR values were derived from RNI using published conversion factors.	Mandatory fortification is associated with higher micronutrient intakes	Survey response rate was low for South Africa. No measured vitamin A level in flour; estimates were based on iron levels.
Folate, iodine, zinc, and other nutrients							
Noor et al., 2017 [25]	600 nonpregnant WRA (18–49 y) living in the Temeke and Ilala districts of Dar es Salaam, Tanzania	A prospective cohort study among WRA enrolled concurrently with the initiation of mandatory fortification.	Compared intakes and plasma folate status of the cohort at 6 and 12 mo of fortification with baseline levels (before initiation of fortification)	Folate intake, plasma folate and fortified foods consumption at baseline, and at 6 and 12 mo after mandatory fortification.	Mean plasma folate concentrations increased from 5.44 ng/mL (±2.30) at baseline to 10.08 ng/mL at 6 mo and 9.70 ng/mL at 12 mo. 25% reduction in the risk of folate deficiency was observed at 12 mo.	Fortification is associated with higher folate status and reduced risk of folate deficiency	The study did not measure fortificant levels in food samples to correlate changes in plasma folate levels with fortification.
Modjadji et al., 2007 [26]	80 nonpregnant WRA (18–44 y) from Capricorn district of Limpopo province, South Africa	A prospective cohort study, comparing baseline postfortification assessments of nutritional status in the same group.	Compared serum biomarkers of nutritional status taken before and 9 mo after mandatory food fortification was introduced.	Serum folate, ferritin, vitamin B_12_, red blood cell folate, and full blood count were assessed.	Median serum and RBC folate, Hb, and hematocrit levels significantly increased after fortification. No change for serum ferritin. Vitamin B_12_ levels decreased significantly. Prevalence of severe and moderate folate deficiency reduced from 16.3% and 11.3% to 0% for both after fortification.	Fortification was associated with higher folate status but not iron status over the 9-mo period.	The study did not measure biomarkers of inflammation which are known to influence serum ferritin concentration as an acute phase protein. The sample size was also small.
Osei et al., 2016 [27]	100 lactating women, mean age = 27.7, from 2 peri-urban settlements in South Africa	Cross-sectional study of potential predictors of urinary iodine concentration (UIC), and breast-milk iodine concentrations (BMIC).	Assessed iodine content of household salt, and measured UIC and BMIC of urine and breast-milk from lactating women and their infants from those households.	UIC, thyroid function, and (BMIC), household SIC	Most women (90%) used adequately iodized salt in the household (≥15 ppm). 39% of mothers had UIC <100 μg/L. SIC predicted both BMIC and maternal UIC.	Adequately iodized salt was associated with higher iodine status and positively predicted breast-milk iodine levels.	The sample size of the study was small
Rohner et al., 2016 [28]	Salt from 1123 households, and urine from 817 nonlactating and nonpregnant women and 154 pregnant WRA (15–49 y) in Sierra Leone.	A nationally representative cross-sectional survey of salt iodization levels and urinary iodine concentrations of pregnant and nonpregnant WRA	Compared household salt iodine intakes with iodine status determined by urinary iodine concentrations.	Adequately iodized salt was defined as iodine ≥15 mg/kg. Adequate UIC for WRA was defined as 100–199 μg/L for nonpregnant and 150–249 μg/L for pregnant women.	80.7% of household salt was adequately iodized. The median UIC among pregnant women was 175.8 μg/L and 203.3 μg/L for nonlactating and nonpregnant women. Women living in households with adequately iodized salt had higher median UIC compared with women living in households with lower iodine content of salt (for pregnant women: 180.6 μg/L vs. 100.8 μg/L, respectively, *P* < 0.05; and for nonpregnant women: 211.3 μg/L vs. 97.8 μg/L, *P* < 0.001).	Consumption of adequately iodized salt was associated with higher iodine status and iodine sufficiency.	The number of pregnant women in the study was well below the minimum sample size of 300 recommended for assessment of UIC [29].
Fereja et al., 2018 [30]	Household salt and urine samples from 356 pregnant women (15–44 y) from rural areas of Ada district, Ethiopia	A community-based, cross-sectional study on salt iodine content and iodine status	Assessed household salt samples to determine iodine concentration and urine samples to measure UIC	Urinary iodine concentration. Household salt iodine concentration. Presence of goiter. UIC is adequate if 150–249 μg/L	Median household salt iodine concentration of the population = 12.2 ppm. Only 39.3% of women consumed adequately iodized salt (≥15 ppm), and 77.6% of pregnant women had insufficient iodine intake. 20.2% prevalence of goiter was found among pregnant women.	Adequate iodine fortification was associated with higher low iodine status.	Study did not control other nutrients like selenium, which may exacerbate the effects of iodine deficiency.

Abbreviations: BMIC, breast-milk iodine concentration; CI, confidence interval; DHS, Demographic and Health Surveys; EAR, estimated average requirements; MAR, mean adequacy ratio; OR, odds ratio; RBC, red blood cell; RNI, recommended nutrient intakes; SIC, salt iodine concentration; WRA, women of reproductive age.

**TABLE 2:** Studies assessing consumer’s acceptability of multiple fortified foods in Africa.

Study reference	Population, sample size and location	Study design	Interventions/assessments	Interventions	Findings	Limitations
Wessells et al., 2024 [31]	Women of reproductive age, ≥15 y (*n* = 84) in Kumbungu and Tolon districts in northern region of Ghana.	Double-blinded, randomized, controlled acceptability study	Acceptability of 2 multiple micronutrient-fortified bouillon cubes, compared with a control cube. Center-based sensory evaluations In-home evaluation of the acceptability and use of study-provided bouillon cubes.	10 g of shrimp bouillon cubes, fortified with 30 mg iodine only. or fortified with a. Vitamin A*—*200 μg RE, folic acid*—*80 μg, vitamin B_12_*—*1.2 μg, iron*—*4 mg, zinc 3 mg, and iodine 30 μg. b. Vitamin A*—*296 μg RE, folic acid*—*28.8 μg, vitamin B_12_*—*0.288 μg, iron*—*1.3 mg, zinc 1.68 mg and iodine 30 μg	There were no statistically significant differences in the mean overall liking among the 3 formulations. 89% of participants rated the 3 bouillon product formulations as “like” or “like very much,” across all evaluations. Fewer participants rated their overall liking of the “upper-level” multiple micronutrient-fortified bouillon cube as “like” or “like very much” (89.2%) compared with the control cube (98.8%).	Data were not collected on intrahousehold distribution of bouillon and were therefore unable to calculate individual intake. Social desirability bias may have led to a possible reluctance among the study participants to give negative ratings regarding the acceptability of the cubes.
Aaron et al., 2011 [32]	32 adults: male and female ≥18 y in Senegal.	Taste and acceptability trial	Acceptability*—*flavor, texture, using a 7-point hedonic scale. Threshold*—*ascending method of limits with 2-of-5 difference tests across 5 concentrations of zinc in breads.	Acceptability test: 3 tests-- a. 1.15 mg iron and 1.5 mg folic acid per kg flour, no zinc.b. Same levels of iron-folic acid as in a. and 63 mg zinc/kf flour. 3. same levels of iron-folic acid as in a. and 126 mg zinc/kg flour. Threshold test: 5 concentrations of zinc in bread (80 mg increments ranging from 80 to 400 mg zinc/kg flour)	Acceptability—high participant ratings, with no significant differences for appearance, flavor, texture, or overall degree of liking. Threshold—no significant differences in degree of liking for all levels of zinc fortification. For all tests, 79%–92% of the participants rated the perceived differences between products as “small” or “by chance”	For the bread threshold test, significant difference was detected for some concentrations in the range and not others, suggesting that the 2-of-5 test may not be the most appropriate for a detection threshold.
Darko, 2010 [33]	60 female (27–50 y) workers at the Vaal Triangle Technikon South Africa.	30 women each assigned to 2 groups, Unfortified Stock Powder and Multiple Fortified Stock Powder (MFSP), respectively.	Acceptability questionnaire on color, smell, taste, and aftertaste of the given stock powder.	Unfortified stock powder or 5 g of multiple fortified stock powder containing 500,000 RE vitamin A, 0.420 mg vitamin B_1_, 0.480 mg vitamin B_2_, 5.400 mg vitamin B_3,_ 0.600 mg vitamin B_6_, 0.134 mg folic acid, 0.300 mg vitamin B_12_, 18.000 mg vitamin C, 4.620 mg iron and 3.000 mg zinc	No significant difference between groups. 72% of MFSP compared with 89% of USP did not detect any color change in their cooked food. No smell or medicinal taste was detected in the food prepared with the MFSP. Very little difference was found between the different powders in terms of aftertaste	No organoleptic changes to the fortified stock powder. Risk of respondents’ exposure to already fortified stock seasoning on market.

Abbreviations: MFSP, multiple-fortified stock powder; RE, retinol equivalents; USP, unfortified stock powder.

**TABLE 3:** Studies assessing consumer’s knowledge and awareness of fortification in Africa.

Study reference	Population, sample size and location	Study design	Outcome assessment	Findings	Limitations
Mwandelile et al., 2019 [34]	698 women (18–49 y) Ifakara Town Council, Kilombero district, Morogoro region, Tanzania.	Community-based cross-sectional study	Awareness of folic acid, awareness of existence of folic acid-fortified flour in community and intake of folic acid-fortified flour. Intake defined as reported consumption of folic acid-fortified flour at least once within 7 d before the survey.	Very low knowledge of folic acid (6.9%). Awareness of folic acid was reported more frequently among women with secondary education or more (13.4%, *n* = 18). Only 7.5% of participants had heard of folic acid-fortified flour. 63.3% consumed folic acid-fortified flour brands.	Possible recall bias.
Motadi et al., 2016 [35]	360 women (16–46 y) in Mopani District, Limpopo Province, South Africa	Descriptive cross-sectional study	Assessed women’s knowledge of food fortification and access to mass media and information regarding fortification using a questionnaire	54% could correctly identify foods fortified in South Africa, 57% knew 1 nutrient added to fortified products. 70% associated malnutrition to fortification, 57% could identify the correct fortification logo on products in South Africa. 57% could define fortification	Knowledge was based on individual question responses, a combined score of responses could better reflect knowledge of the population studied.
Mabaya et al., 2010 [36]	452 participants Males (149) and females (303) Ages 30–70 y in Gaborone and southeast districts of Botswana	Cross-sectional	Consumer preferences assessment (1–10) based on product attributes of brand, quality, price, color/appearance, nutritional value, made in Botswana. Consumer knowledge and willingness to pay extra for fortified foods	Desired product attributes by consumers were brand, quality, price, and color or appearance, all scored a mean of >7. Health or nutritional quality was scored 6.3. 40% had limited knowledge and 2% knew a lot. 70% were not willing to purchase fortified foods at a higher price	Poor knowledge of fortification among respondents may have confounded their preference and willingness to purchase fortified foods.

## Results

Thirteen primary studies met the inclusion criteria for assessment of the effects of food fortification on nutritional status or health biomarkers for WRA in Africa. Four studies were conducted among women in South Africa [20,21,26,27], 2 in Tanzania [18,25], and 1 each in Cote D’Ivoire [23], Sierra Leone [28], Ethiopia [30], Cameroon [19] and Ghana [17]. One study included survey data from 4 sub-Saharan African countries, namely Nigeria, South Africa, Tanzania, and Uganda [24], and 1 estimated the changes in anemia prevalence across 9 African countries with mandatory fortification regulations [22]. All the studies were published between 2003 and 2024.

The included studies evaluated the effectiveness of fortification on nutritional status and serum biomarkers, using different study designs such as double-blinded controlled trials [17,18], pre–post prospective cohort design [19,20,25,26], and cross-sectional cluster or household surveys [23,24,27]. One study estimated the changes in nutrient intakes through fortification by substituting unfortified products in secondary dietary intake data for fortified foods with known micronutrient contents determined through analysis of market samples of fortified products [21]. Another study used Demographic and Health Survey data from multiple African countries to compare trends in anemia prevalence for countries with or without mandatory food fortification.

Serum biomarkers of nutritional status including plasma concentrations of hemoglobin, ferritin, transferrin, retinol-binding protein, and plasma folate were measured and compared for changes following either targeted or mandatory fortification in several of the studies. These were useful for estimating iron, vitamin A, and folate deficiency or risk of anemia in the study population based on predefined thresholds for nutrient sufficiency and deficiency. Concerning iodine fortification and nutritional status, 3 studies assessed household salt iodine concentration and breast-milk or urinary iodine levels of lactating women and pregnant or nonpregnant women to estimate iodine status [27]. Some studies measured biomarkers of inflammation to explain the effects of fortification on iron status. For nutrient intake status, the daily mean intakes of vitamin A, iron, iodine, and folate were reported by some studies. The details of the study population, design, assessments, and findings from all the studies have been tabulated in Table 1.

### Effects of iron and vitamin A fortification on plasma biomarkers, nutrient intake, and nutritional status of women

Mixed outcomes of the effects of fortification were reported by studies on serum biomarkers for iron and vitamin A [17,18]. In a high anemia prevalence population of northern Ghana, a double-blinded randomized trial found that consumption of multiple micronutrient-fortified biscuits containing 11 added vitamins and 7 minerals was associated with no impact on vitamin A status of 309 adolescent girls when compared with girls consuming unfortified biscuits (*n* = 312) after 28 wk [17]. Vitamin A status was measured as retinol-binding protein concentration in this study. The study also found no difference in hemoglobin and iron status following the 28-wk trial between the 2 groups, although the multiple micronutrient-fortified biscuits contained 80% more vitamin A and 75% more iron than the levels in the unfortified biscuits.

A stratified analysis of the data, however, showed that baseline vitamin A deficiency, 33%, and menarche status of the girls influenced the intervention's effect on vitamin A status. A significant increase in retinol-binding protein concentration (12.6%) and a 9.6% reduction in low or deficient vitamin A status was found for the subgroup of postmenarche girls in the micronutrient-fortified biscuits group compared with those who consumed the unfortified biscuits. No difference was, however, observed in hemoglobin levels and anemia prevalence after the subgroup analysis [17]. These findings highlight the confounding effects of individual factors such as development and baseline nutritional status on the impact of fortification.

Similarly, among 2 groups of pregnant women in a randomized placebo-controlled double-blind trial, there was no improvement in vitamin A status and no difference in retinol concentration between the groups after 8 wk of micronutrient-fortified beverage consumption [18]. For this study, participants either consumed a micronutrient-fortified beverage containing 11 micronutrients or an unfortified beverage daily and effects on serum vitamin A and iron biomarkers were compared with baseline levels after 8 wk of beverage consumption [18]. The study found that consuming the fortified beverage resulted in a 4.16 g/L increase in hemoglobin concentration and a 3 μg/L increase in ferritin compared with the unfortified beverage group. The risk of anemia and iron deficiency anemia in the fortified beverage group was reduced by 51% and 56%, respectively. No change was, however, found in risk of anemia and iron deficiency anemia among the pregnant women in the control group [18].

The change in hemoglobin concentration in the group consuming the fortified beverage can be attributable to a combined effect of other micronutrients, such as folic acid and vitamin B_12_, and not just iron, in the fortified beverage, contributing to an improved hemoglobin level by correcting other forms of nutritional anemia. This potential effect was, however, not observed among the adolescent girls in the Ghana study who received similar multiple micronutrients in the fortified biscuits. The already high prevalence of iron deficiency among the adolescent girls in the Ghana study may have limited the impact observed after consuming fortified biscuits [17]. The iron levels in the fortified biscuits in the Ghana study were also not high enough to meet the iron requirements of the girls and overcome deficiency [17].

A pre–post study using 2 surveys, 2 y before (2009) and 1-y after fortification (2012), assessed the changes in iron, folate, zinc, and vitamin B_12_ status of WRA in Cameroon with mandatory wheat flour fortification [19]. In this study, >90% of the respondents reported the consumption of wheat flour in the previous week, suggesting good exposure to the food vehicle and potentially to the intervention (adequately fortified flour). Compared with the prefortification nutrient intakes, the prevalence of inadequate iron intake was found to be lower, 66% compared with prefortification prevalence of 85%. Inadequate zinc intake in the population was also lower, 13% after fortification compared with prefortification inadequate intakes of 40%. Folate intake below the estimated average requirements (EAR) was lower by 66 percentage points (79%–13%), and the prevalence of anemia was lower, 39.1% after fortification compared with prefortification levels of 46.7%. However, no change in mean maternal hemoglobin concentrations was found between the pre- and postfortification periods [19]. Despite a good exposure to wheat flour in the study population, resulting in a lower prevalence of inadequate micronutrient intakes, the levels of inadequacy remaining in the postfortification period were still notably high, needing further mitigation [19].

Similarly, a prospective cohort study among lactating women in South Africa compared nutrient intakes and serum concentrations of retinol, folate, ferritin, and zinc before and after the initiation of mandatory fortification. The measurements of serum parameters, which were taken 6, 14, and 24 wk and 9- and 12-mo postpartum, indicated that dietary intakes postfortification were higher for all fortified nutrients, with significantly higher serum folate and zinc compared with levels before fortification. However, there was no change in ferritin levels and retinol levels were lower for the women postfortification [20]. The estimated dietary iron intake postfortification was higher with all the women exceeding the EAR for iron, but there was no association observed between the higher iron intakes and the prevalence of iron deficiency among the women after fortification [20].

A secondary data analysis, including 1726 WRA in South Africa after the initiation of mandatory flour fortification, found that consumption of analyzed fortified samples of maize flour and fortified white and brown bread was associated with increased micronutrient intakes of women, resulting in higher mean adequacy ratio achieved, 72.7% compared with 59.9% for consumption of unfortified products. Fortified flour consumption was also associated with an increased proportion of women meeting 100% of their micronutrient requirements for all nutrients except iron and folate [21]. Lower levels of iron and folate fortification of flour than are required by the fortification standards of the country may be a reason for the lack of impact observed.

Rondini et al. [22] compared the change in anemia prevalence for countries with and without mandatory food fortification programs, based on nationally representative data from 2 or more Demographic and Health Surveys completed between 2000 and 2018. They found that countries with mandatory flour fortification showed 27% decreased odds of anemia and a 7.5% decrease in the mean anemia prevalence among nonpregnant WRA from the pre- to postfortification period, compared with countries without mandatory food fortification regulations.

The quality and level of fortification of flour were also identified by Rohner et al. [23] as factors that limit the effects of iron fortification on micronutrient status of WRA living in rural and urban areas of Côte d’Ivoire. They found significant differences in the coverage of adequately fortified flour between the rural (47%) and urban (93%) areas through assessing the micronutrient contents of wheat flour samples. They further found that among nonpregnant WRA selected from 26 rural and urban clusters in Côte d’Ivoire, the mean hemoglobin concentrations were significantly lower among rural than urban WRA. More than 3-quarters of the rural WRA were found to be anemic, compared with less than half of the urban sample [23].

Similarly, a cross-sectional, clustered household survey found that suboptimal iron fortification levels of mandatory fortified foods limited the contribution of fortification to iron requirements of WRA in Nigeria and South Africa (subnational population) and in Tanzania and Uganda (national population) [24]. Evaluating how measured micronutrient levels in fortified maize and wheat flour, oil, and salt in households was associated with nutrient intakes and requirements of WRA, the authors found that the estimated daily micronutrient intakes from fortified foods contributed substantially to the requirements of WRA for vitamin A and iodine (20%–125% and 88%–253% EAR), respectively, but not for iron [0%–13% recommended nutrient intakes (RNI)]. They suggested that if recommended levels of fortification were achieved, the potential iron intakes from flour fortification would be higher and meet ≤65% of the RNI for WRA in these 4 countries [24].

### Effects of folate, zinc, and iodine fortification on nutrient intake and nutritional status and serum biomarkers

Significant changes in serum biomarkers and nutritional status for folate, zinc, and iodine were reported by studies comparing micronutrient status of women before and after fortification in Africa. Noor et al. [25] evaluated the potential of folate fortification to change serum folate status and the prevalence of folate deficiency among 600 nonpregnant WRA living in 2 districts in Dar es Salaam, Tanzania. They used a prospective cohort design and enrolled nonpregnant women concurrent with the initiation of mandatory flour fortification and followed up for 1-y thereafter. Comparing the change in plasma folate levels at 6 and 12 mo of the fortification program from baseline, they found higher mean plasma folate concentrations, 10.08 ng/mL at 6 mo and 9.70 ng/mL at 12 mo, compared with 5.44 ng/mL at baseline. The prevalence of folate deficiency was also lower, 5% at 12 mo of fortification than 26.9% at baseline, based on a plasma folate cut-off level of 4 ng/mL. There was also a 25% lower relative risk of folate deficiency at 12 mo for every 1 ng/mL increase in plasma folate from baseline levels [25].

Similarly, a prospective cohort study in South Africa among 80 nonpregnant WRA, beginning before the mandatory flour fortification program in 2003 and ≤9 mo post initiation found that median levels of serum folate, red blood cell folate, hemoglobin, and hematocrit levels were significantly higher among the women after fortification [26]. The prevalence of severe and moderate folate deficiency was lower, 0% in both cases compared with 16.3% and 11.3%, respectively, before fortification. Similarly, low red blood cell folate concentration was significantly lower, 1.9% compared with 26.4% before fortification. However, no change was observed for serum ferritin and hemoglobin levels over the period of fortification [26]. These 2 studies [25,26] highlight the potential of folate fortification to address deficiency relatively quickly.

For iodine fortification of salt, the effects of consumption of iodized salt and other iodine-containing foods on breast-milk iodine concentrations and urinary iodine concentration (UIC) of breastfed infants were assessed among 100 lactating women from 2 peri-urban settlements in South Africa [27]. It was found that consumption of iodized salt was the main predictor of optimal breast-milk iodine concentrations and UIC of the breastfed infants and their mothers.

Similarly, a nationally representative cross-sectional survey in Sierra Leone assessed household salt iodine and UIC of 817 nonpregnant and 154 pregnant women and found that women living in households with adequately iodized salt had higher median UIC compared with women in households with low iodine content of salt [28]. Although the study did not estimate the prevalence of iodine deficiency in the population, it found that median UIC among households with adequately iodized salt was adequate and significantly higher for pregnant women compared with women in lower iodized salt households, suggesting iodine sufficiency as a positive effect of consuming adequately iodized salt [28].

Among 356 pregnant women in rural areas of Ethiopia, poor quality of salt iodization was associated with an increased risk of goiter and increased prevalence of iodine deficiency [30]. This community-based cross-sectional study found a 20.2% prevalence of goiter among pregnant women, with a median household salt iodine concentration of 12.2 ppm (IQR: 6.9–23.8), lower than the threshold for adequately iodized salt. The median UIC of the pregnant women in the study was also 85.7 μg/L and 77.6% of them had a UIC below the recommended 150 μg/L, "suggesting iodine sufficiency may be a positive effect of consuming adequately iodized salt" [30].

### Assessment of knowledge and acceptability of fortification among WRA in Africa

Three studies (Table 2) used a randomization design to assign women to groups for evaluating the acceptability or degree of liking of varying micronutrient levels in fortified products based on sensory characteristics: taste, smell, and color [[31], [32], [33]]. The Ghana study evaluated the acceptability of 2 multiple micronutrient-fortified bouillon cubes compared with a control cube using a double-blind, randomized controlled design [31]. An experimental study design was also used to evaluate compliance and consumer acceptability of a multiple fortified stock powder compared with unfortified stock powder among 2 groups of 30 women each in South Africa [33]. In the Senegal study, acceptability tests were performed on bread at varying degrees of fortification with iron, folic acid, and zinc [32]. Three studies also evaluated knowledge, awareness, and preference of fortification and fortified products among consumers using cross-sectional surveys [[34], [35], [36]] (Table 3).

### The degree of liking and acceptability of multiple fortified foods

There was an overall liking for all multiple fortified products among consumers in the 3 studies and higher levels of fortification did not affect the taste, color, and overall acceptability of the products. The overall liking of 3 bouillon cube formulations, fortified with iodine only (control), with higher amounts of multiple micronutrients, (vitamin A, folic acid, vitamin B_12_, iron, zinc, and iodine) and with lower amounts of the same multiple micronutrients was evaluated among 84 WRA and their households in Ghana [31]. Using a double-blind, randomized, and controlled approach, women from 2 peri-urban communities of northern Ghana were presented with dry fortified bouillon cubes and meals prepared with them for assessment of their acceptability. There were no statistically significant differences in the mean overall liking of the 3 formulations of fortified bouillon cubes. Acceptability was assessed based on physical and sensory characteristics, such as appearance, feel/texture, crumble, smell/aroma, taste, and overall acceptability. Eighty-nine percent of the participants rated their overall liking of the 3 bouillon formulations as “like” or “like very much,” and within this group the high-level multiple fortified bouillon cube was only slightly less popular (89.2% positive rating) compared with the control cube (98.8%). No differences were found with participants’ evaluations of the overall acceptability of the different bouillon cube formulations when they were blindly assigned with cubes for use in their household dishes for 14 d.

Similarly, Darko [33] found no significant difference in acceptability of multiple fortified stock powder containing iron, folic acid, vitamin A, zinc, vitamin C, and vitamins B_1_, B_2_, B_3_, and B_6_ when compared with unfortified stock powder by participants in a small experimental study involving 60 WRA in South Africa. Acceptability was evaluated based on women’s preference for the color, smell, taste, and after taste of the assigned stock powder, multiple fortified or unfortified.

A zinc-fortified bread acceptability and zinc threshold trial conducted among 32 adults in Senegal also found no difference in the pooled mean for overall degree of liking of 3 bread samples fortified with 1.15 mg iron as ferrous fumarate and 1.5 mg folic acid per kg flour only, or with additional 63 mg or 126 mg zinc as zinc oxide per kg flour. No difference in acceptability was found for individual assessments of appearance, flavor, or texture [32]. For all tests, 79%–92% of the participants rated the perceived differences between products as “small” or “by chance.” However, the authors found that significant differences were detected for some concentrations by participants in the zinc threshold tests when zinc fortification of bread was done at 80 mg increments ranging from 80 to 400 mg zinc oxide per kg flour [32].

### Factors that determine consumer awareness and consumption of fortified foods in Africa

Three studies, assessing consumer knowledge and perceptions of fortification in Africa, were reviewed. These studies used questionnaires to assess consumer awareness or knowledge of fortification. One study included both male and female respondents in the survey. Using a cross-sectional survey, Mabaya et al. [36] assessed consumer purchasing behavior, attitudes, knowledge, and willingness to pay extra for fortified products in Botswana, and found that the most important product attributes for consumers were the brand, quality, price, and color or appearance. Each of these attributes received a mean score of 7 on a scale of 1–10. The health impact of the product scored a mean of 6.3. In this population of 452 adults aged 30–70, 58% knew nothing about fortified foods, 40% reported limited knowledge, and 2% knew a lot. The majority of the participants (>70%) answered “no” to survey questions examining their willingness to purchase at a higher cost or pay more for fortified products. Only a few respondents were willing to pay up to an additional 5% of the cost of unfortified products for a fortified alternative.

Similarly, a survey among South African WRA found that only half of the women had some knowledge of food fortification in the country. About 54.0% could correctly identify foods fortified in South Africa, and 57% knew 1 micronutrient added to fortified products. The majority (70%) were able to identify fortification as an intervention for malnutrition, and 57% could identify the correct fortification logo on products in South Africa [35]. This outcome suggests that nearly half of the women did not know food fortification in a country where mandatory food fortification has been implemented for nearly 50 y [35].

The Tanzania study also evaluated women’s knowledge, awareness, and consumption of folic acid-fortified flour through a community-based cross-sectional study [34]. The authors found that women’s knowledge of folic acid was very low (6.9%) and only 7.5% of the participants had heard of folic acid-fortified flour. However, 63.3% had consumed folic acid-fortified flour brands at least once within the 7 d before the survey. Exploring some factors associated with knowledge of folic acid fortification, they found that women with secondary education or more (13.4%) reported awareness of folic acid more frequently and the odds of folic acid-fortified flour intake within the 7 d before the survey were 2 times higher for employed women compared with unemployed women [34].

## Discussion

This review provides a summary of studies assessing the effects of large-scale and targeted (small-scale) food fortification on nutrient intakes and the nutritional status of WRA in Africa. It also provides a snapshot into consumer acceptability and knowledge of multiple-fortified foods in Africa. To the best of our knowledge, this study is the first to synthesize outcomes of nutritional status and micronutrient deficiencies from food fortification efforts for WRA in Africa. Few studies have evaluated the effects of food fortification on nutrient intake, nutritional status, and the prevalence of micronutrient deficiencies among WRA in Africa. The studies we reviewed explored micronutrient levels of fortified food samples in households or based on the nationally recommended levels of fortification and compared them with changes in serum or other biological biomarkers for nutritional status. We found that food fortification positively improves the intake of micronutrients but not the nutritional status of WRA for some micronutrients in Africa [19,20,[23], [24], [25], [26]].

### Effects of iron and vitamin A fortification on nutrient intake, plasma biomarkers, and nutritional status of women

Fortification with iron and vitamin A yielded variable outcome on nutritional status in this review. We found that although food fortification was associated with higher iron and vitamin A intakes, changes in nutritional status and serum biomarkers were significantly lower for WRA in Africa [19,20,[23], [24], [25], [26]]. Longer-term observational studies assessing large-scale fortification programs found that fortification was associated with increased intakes and better nutritional status for iron and vitamin A. Anemia prevalence was found to be lower over the years for countries with mandatory fortification compared with countries without mandatory fortification. This change in anemia prevalence could have resulted from the collective effects of other micronutrients, such as folate, vitamin A, and vitamin B_12_ in the fortified products, on nutritional anemias but also as a result of the long-term higher iron intakes from mandatory fortification compared with the trials with short duration or the 1-y prospective cohort studies that found no change in anemia prevalence [1720]. Intervention studies seem to find an improvement in iron and vitamin A intakes but not necessarily a change in status [[17], [18], [19],^28^]. This is likely because these interventions were just too short for a change in nutritional status to be observed and the interventions may also have provided too little micronutrients in the fortified products compared with what is in the control food; hence, no change in nutritional status was observed for the groups receiving the intervention. In the case of iron fortification, not having a criterion for ruling out non-nutritional anemias may have resulted in the lack of fortification effects observed on anemia prevalence. The multiple micronutrient contents of fortified foods and the interactions between these micronutrients could have also confounded the observed outcomes of fortification on nutritional status in the intervention studies.

Vitamin A fortification was associated with a change in nutritional status only when factors such as baseline deficiency of the population were controlled. In this same population with high baseline vitamin A deficiency, iron fortification had no effects on hemoglobin and anemia status, corroborating findings from a systematic review that found a positive effect of adequate vitamin A status on serum ferritin and hemoglobin levels [37]. Although food fortification has improved the availability of essential micronutrients such as iron, iodine, and vitamin A in many regions worldwide [38], African countries would benefit from complementing this strategy with effective disease control measures and periodic multiple micronutrient supplementation for vulnerable groups, such as adolescent girls and WRA. This integrated approach can help mitigate the inhibitory effects of widespread micronutrient deficiencies and infectious diseases on the effectiveness of food fortification programs.

The limited effects of iron fortification on iron status may be attributed to the quality of fortification and the choice of iron fortificants. In particular, the poor bioavailability of certain iron compounds used in flour fortification by millers could be a contributing factor. Additionally, diets in LMIC including Africa are predominantly plant-based and rely heavily on cereal-based staples, which are high in phytates [8]. These compounds inhibit the absorption of less bioavailable forms of iron, further reducing the effectiveness of fortification efforts. Hurrell et al. [39] found that a significant number of mills in developing countries use nonrecommended iron compounds with poor bioavailability for grain flour fortification, mainly because these forms of iron fortificants produce no or less noticeable sensory changes in taste and color of fortified flour. Some mills also fortify flour with lower levels of the iron compounds than is recommended by the country’s fortification standards [39]. The global fortification data exchange estimated that <50% of the 72 countries with mandatory flour fortification meet the WHO nutrient-level recommendations for iron fortification [16].

These gaps in fortification result in a lower or zero magnitude of change in nutritional status among women consuming such fortified foods [20,24]. As recommended by the WHO, relevant stakeholders in African countries with flour fortification programs can improve the impact of fortification by establishing robust monitoring structures to regulate and ensure the quality of fortified products [40,41]

Inequalities in the availability and coverage of adequately fortified foods across vulnerable populations also affect the outcome of fortification on nutritional status in the same population [42]. This review found lower hemoglobin status and a higher prevalence of anemia among nonpregnant WRA in rural areas compared with their urban counterparts as a result of the consumption of wheat flour which had significantly lower levels of iron in rural areas compared with the availability of adequately iron-fortified wheat flour in urban areas, [23].

Mkambula et al. [43] reported this inequality gap in their study which found that across 16 LMIC with wheat flour fortification programs, nearly 40% of households are not reached with micronutrients, because some industrially milled flours are either unfortified or fortified at levels below the recommended standards. These gaps were more pronounced for vulnerable populations including, rural dwellers, poorer households, and households with poor women's dietary diversity than for nonvulnerable populations like urban dwellers [42,44]. Fortification programs must be regulated and monitored in such a way that all consumers, both in rural and urban settings, have equitable access to the same quality of adequately fortified foods and at a constant supply to ensure adequate delivery of micronutrients to the most deficient populations.

### Effects of fortification on folate, zinc, and iodine intakes among WRA in Africa

We found that folate, zinc, and iodine fortification of food in Africa improves the intakes and nutritional status of WRA exposed to fortified food products. It is unclear, however, whether these changes in nutritional status are sustained after longer periods of establishing mandatory food fortification programs as the majority of these studies evaluated the changes within the first 2 y of initiating fortification with no further follow-ups. Iodized salt consumption among WRA was associated with adequate iodine status and met estimated requirements for WRA living in households with access to adequately iodized salt in this review. Salt iodization in itself is an effective intervention for the prevention of goiter and other iodine deficiency disorders. A systematic review by Keats et al. [45] estimated that salt iodization led to a 74% reduction in the odds of goiter and prevention of mental retardation and other subclinical iodine deficiency disorders in LMICs.

Some of the studies reviewed reported lower iodine content in household salt, ≤15 mg/kg, and were associated with lower UIC in the population. In 1 such study, a 20% prevalence of goiter was found among WRA with lower household salt iodine content, [30]. Although this study did not compare UIC between women consuming inadequately and adequately iodized salt for a difference in goiter prevalence, iodine intake below recommended levels is a likely contributor to the goiter risk. Knowles et al. [44] reported that low compliance to iodization requirements and persistent inequalities in household access to adequately iodized salt put almost half of households consuming iodized salt in LMIC at risk of iodine deficiency. Other factors, such as excessive consumption of goitrogens, may also play a role in the risk of goiter.

These findings of lower salt iodine contents than recommended highlight gaps that could be further investigated. Strengthening of regulatory monitoring processes for mandatory salt iodization programs will ensure improved compliance to iodization standards and in cases where low salt iodine is as a result of iodine losses from poor handling practices and exposure of iodized salt to adverse conditions at the point of sale and in households, consumer education could be implemented to promote salt iodine retention.

Folic acid fortification of flour was also associated with a positive change in serum folate status and the reduction in folate deficiency risk among WRA within a year of initiation of fortification [25,26], corroborating findings of the effectiveness of folic acid fortification in the reduction of folate deficiency and the prevention of folate-related disorders including neural tube defects in high-income countries [46].

### Acceptability and awareness of food fortification and vehicles by consumers

The few acceptability trials in this review show that consumers in Africa consider novel fortified food vehicles such as bouillon cubes acceptable for use in household meals. Perceived sensory changes due to higher levels of fortificants were, however, noticeable, highlighting the need for fortified food producers to consider these organoleptic changes when selecting new food vehicles and choosing fortificants. Identifying a suitable vehicle that is widely consumed by the target population, including vulnerable groups such as rural dwellers and is centrally processed for LSFF, remains a challenge in LMICs. This difficulty stems from socioeconomic barriers and the fragmented nature of the food processing industry in these regions. Findings of the acceptability of multiple-fortified stock powders and bouillon cubes suggest they could serve as vehicles for micronutrient fortification, reaching a large number of African consumers.

A study by the Global Alliance for Improved Nutrition supports the suitability of bouillon cubes for LSFF with multiple micronutrients in Burkina Faso and Nigeria, citing their widespread availability, household coverage, and consumption in fortifiable forms as factors that promote their suitability for large-scale fortification. [47]. Surveys in some West African countries found that 79%–99% of WRA consumed 2–3 g of bouillon cubes daily [47,48]. In Cameroon, median intake levels were ∼1.9 g/d for women and 0.9 g/d for children [49].

Because only a few studies in Africa have evaluated consumer acceptance and the feasibility of bouillon cube fortification, their positive findings may not fully reflect overall consumer interest and acceptance across the continent. If African countries were to adopt this approach as part of LSFF efforts, further research would be needed to assess coverage, consumption patterns, and impact. Additionally, studies should explore the feasibility of multiple fortification strategies through the region’s limited number of industrial producers of bouillon cubes, stock powders, and other condiments.

### Consumer awareness and consumption of fortified foods

We found that despite the presence of mandatory food fortification programs, many consumers in some African countries had little to no knowledge of these programs or their benefits. One advantage of this lack of awareness is that, if fortified foods have widespread coverage and come at no extra cost, people will consume essential micronutrients without bias or the need for behavioral change. However, in countries where fortified food coverage is low or manufacturers fail to comply with fortification standards, consumer awareness becomes crucial. Educating consumers about the benefits of fortification and how to identify fortified foods can increase demand for these products. This, in turn, may pressure noncompliant manufacturers to adhere to fortification requirements to remain competitive. In the studies reviewed, the majority of respondents to fortification awareness surveys were WRA, a group often responsible for household meal decision making. Having little awareness of fortification and a poorer preference for fortified foods suggests that many may not be receiving additional nutrition through this complementary strategy if foods were not mandatorily fortified. Such is the case with the study by Mwandelile et al. [34] who found that despite the low awareness of folic acid fortification, the availability of folic acid-fortified flour in the population meant the majority of women were consuming flour with additional micronutrients unknowingly. Empowering women with knowledge regarding food fortification and its effects on MND will positively influence their choices and exert spillover effects on other members of their households. This could be achieved through targeted nutrition education interventions for women at child welfare and antenatal services and through the use of mass communication media.

### Strengths and limitations of this study

This study synthesizes evidence of the effectiveness of food fortification programs, implemented at different scales, on improving nutrient intakes and nutritional status of WRA in Africa. This study also highlights some implementation factors that could potentially result in improved nutritional status at the population level, particularly for large-scale fortification programs and identifies some factors that confound the effects of fortification on nutritional status such as a high baseline prevalence of multiple deficiencies in the population. We included both small intervention studies and observational studies based on country-wide mandatory fortification programs to highlight the potential differences in large-scale and targeted fortification on nutritional outcomes.

This review has some limitations. The literature search focused on 5 commonly deficient micronutrients in Africa, and so findings did not capture other micronutrients that are often added to foods as fortificants such as Thiamine and Niacin. Other limitations stem primarily from the design of the included studies. Most studies used a pre–post design to assess the effects of food fortification on nutritional status, which limits causal inferences, and the observational studies lacked a comparison group, as a result, changes observed in nutrient intake and nutritional status cannot be solely attributed to fortification. Potential confounders, such as shifts in dietary diversity, access to micronutrient supplementation, and the effects of inflammation, may have influenced the outcomes in the study population. Although statistical analyses accounting for these confounding factors could increase confidence in the role of fortification, some uncertainty would remain.

Additionally, some studies assessing nutrient intake and adequacy based on comparison to estimated average requirements did not take into consideration the distribution of iron requirements for WRA, 15–49 y. This may have overestimated the degree of adequacy achieved for younger WRA who may have higher iron intake requirements than the older end of the age range. Despite these limitations, the findings on nutritional status and deficiency prevalence after the introduction of fortification warrant attention.

The majority of studies included in this review were conducted on subpopulations within specific countries, meaning their findings may not be fully representative of the impact of fortification across the entire African population. Similarly, only a limited number of studies examined the acceptability and knowledge of fortification, restricting the generalizability of conclusions to the surveyed populations. Additionally, the reviewed studies did not assess the potential risk of exceeding micronutrient intake requirements or reaching tolerable upper limits due to fortification, leaving micronutrient toxicity unaddressed in this review.

In conclusion, this review found that food fortification with micronutrients enhances the intakes of iron, vitamin A, folate, iodine, and zinc among WRA in Africa and was associated with improved nutritional status and reduction of the risk of deficiency among WRA for folate, iodine, and zinc. Effects of fortification on iron and vitamin A status were however variable depending on the scope, duration, and design of the study. Iron fortification has the potential to impact iron status if key implementation challenges such as noncompliance with standards and the high prevalence of infectious diseases in the population are effectively addressed. As a cost-effective public health strategy for preventing MND at the population level, food fortification could significantly benefit WRA in Africa, who experience a high burden of multiple micronutrient deficiencies.

Currently, there is insufficient data on the effectiveness of food fortification for addressing the range of MND common among WRA in Africa. Governments must strengthen their commitment to effective micronutrient fortification by establishing robust regulatory monitoring systems to ensure that fortification policies translate into actively implemented interventions. Additionally, there should be well-established systems for routine evaluation of food fortification programs for their effects on nutritional status and overall performance. This will provide relevant data for assessing the cost effectiveness and for decision making by policymakers toward refining fortification strategies to maximize the benefits although minimizing the risks.

To enhance coverage, African countries could also explore targeted fortification strategies, including the fortification of multiple food vehicles through small-scale food producers. This approach could be particularly beneficial in regions where fragmented food production limits the reach of large-scale fortification, ensuring that vulnerable populations, especially those in rural areas, receive adequate micronutrient support.

## Author contributions

The authors’ responsibilities were as follows – JBC: analyzed the results and wrote the paper; and all authors: responsible for the design and review of the manuscript, read and approved the final manuscript.

## Funding

JBC is currently a PhD Fellow with the Sustainable Nutrition Initiative and receives her PhD scholarship from the Riddet Institute, Massey University.

## Conflict of interest

The authors report no conflicts of interest.
